# Supplementary material for: Insights into plastocyanin–cytochrome b6f complex formation: The role of plastocyanin phosphorylation
Source: Plant Physiol. 2025 Jun 24;198(4):kiaf269. doi: 10.1093/plphys/kiaf269 (PMC12341890; doi:10.1093/plphys/kiaf269)
Supplement: kiaf269_Supplementary_Data [file kiaf269_supplementary_data.zip › Supplementary data.pdf]

Supplementary Data

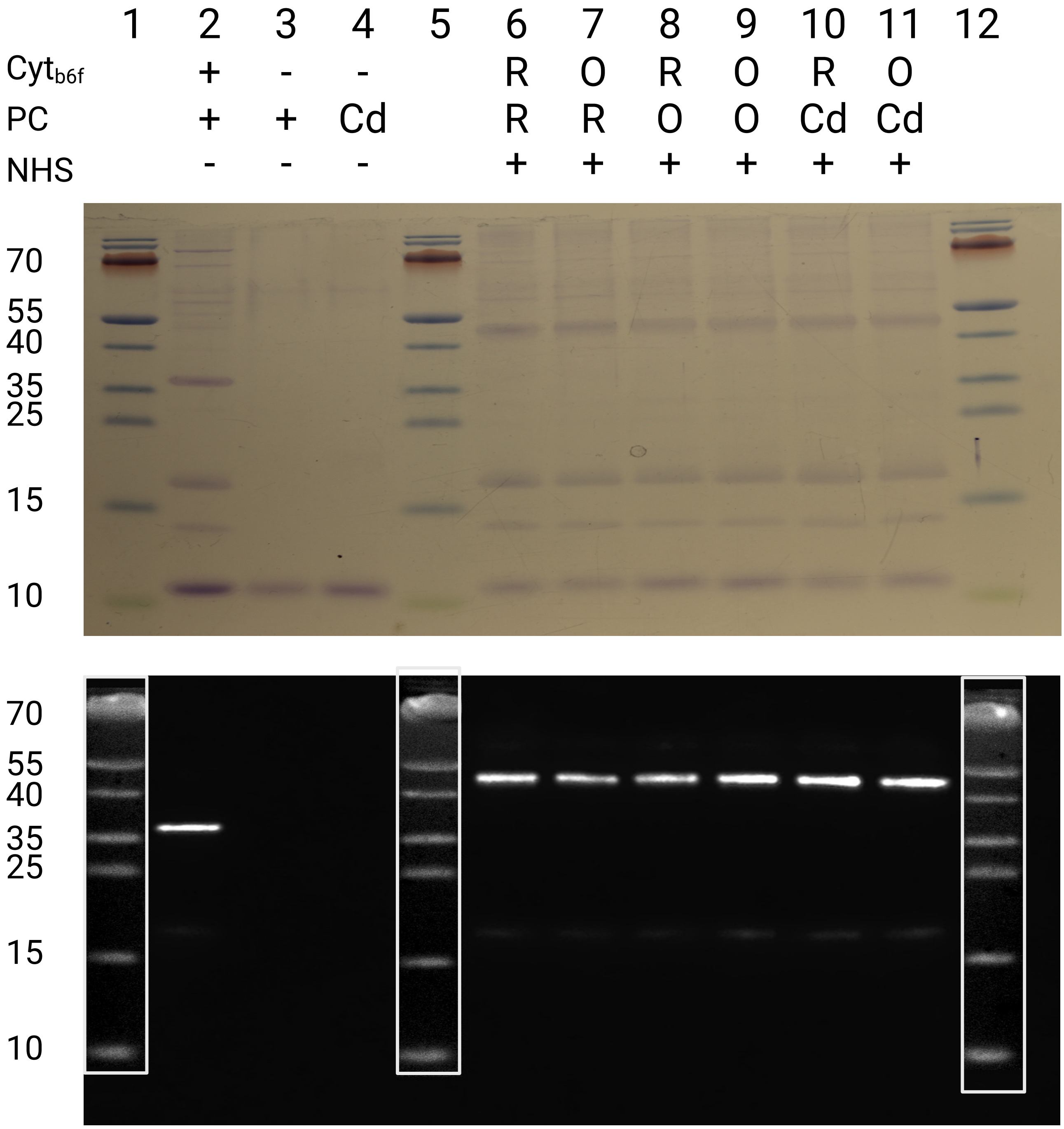

Supplementary Figure S1: Full SDS-PAGE results of cross-linked cytochrome *b_6f* and PC. Recombinant plastocyanin (PC) was either oxidized (O) using $K_3Fe(CN)_6$, reduced (R) using ascorbate or had its copper cofactor replaced by Cadmium (Cd) and activated with 1-Ethyl-3-[3-dimethylaminopropyl]carbodiimid-Hydrochlorid (EDC) and Sulfo-N-Hydroxysulfosuccinim (NHS) (NHS +). It was then mixed with either oxidized (O) or reduced (R) purified Cyt*b_6f*. Samples were loaded on SDS-PAGE and either stained by Coomassie blue (Upper panel) or blotted to a nitrocellulose paper and incubated with anti-Cyt*f* antibodies (Lower panel). First three lanes (left) were added as controls in order to verify that the band shift resulted from the cross-linkers. Size markers (white boxes) were overlaid using the system's software following acquisition. Lanes nr. 1,2 and 6 were used to generate Figure 2a.

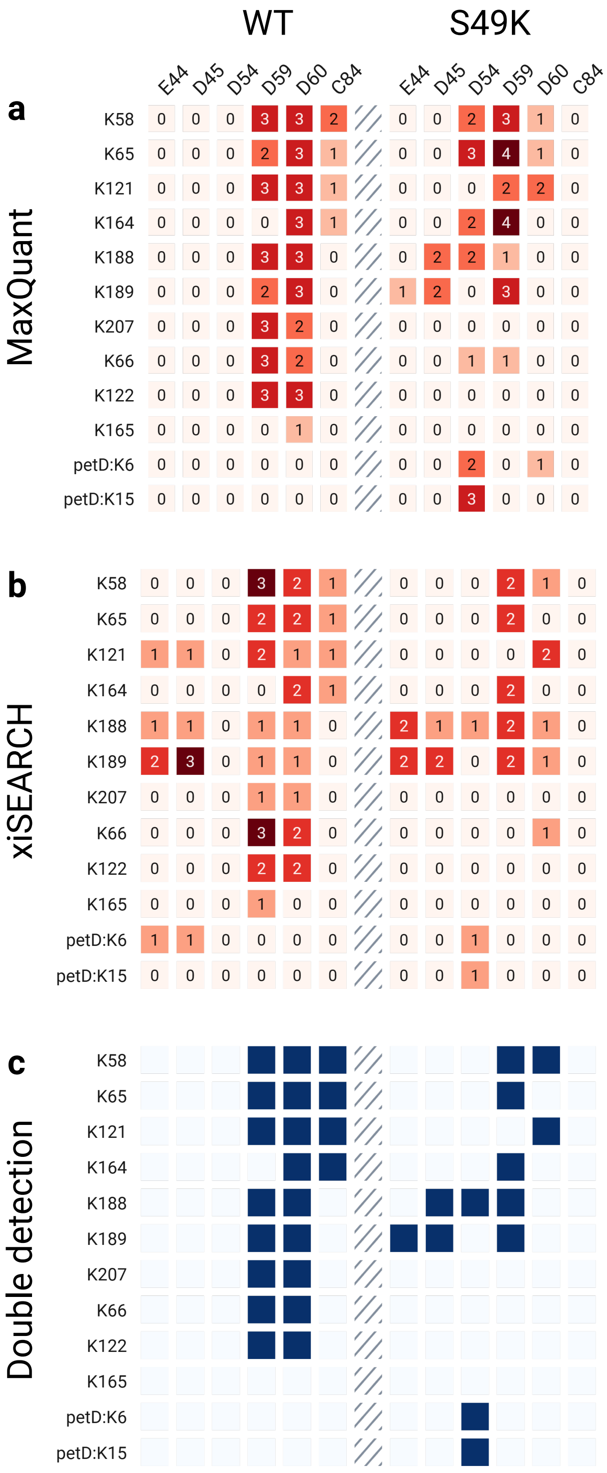

Supplementary Figure S2: EDC-NHS cross-linking of cytochrome *b_6f* and PC. Recombinant plastocyanin was activated with 1-Ethyl-3-[3-dimethylaminopropyl]carbodiimid-Hydrochlorid (EDC) and Sulfo-N-Hydroxysulfosuccinim (NHS) and cross-linked with purified His-tag Cyt*b_6f*. Cross-linked samples of wildtype (WT) and PC mutant (S49K) strains were digested by trypsin and analysed via mass-spectrometry. Data was analyzed using both MaxQuant (**a**) and XiSearch (**b**) algorithms. The resulted cross-linking events were compared, where positive results are marked in blue (**c**) and peptides that were detected in using both algorithms were taken into consideration in the modeling restriction. The illustration was generated using BioRender.com

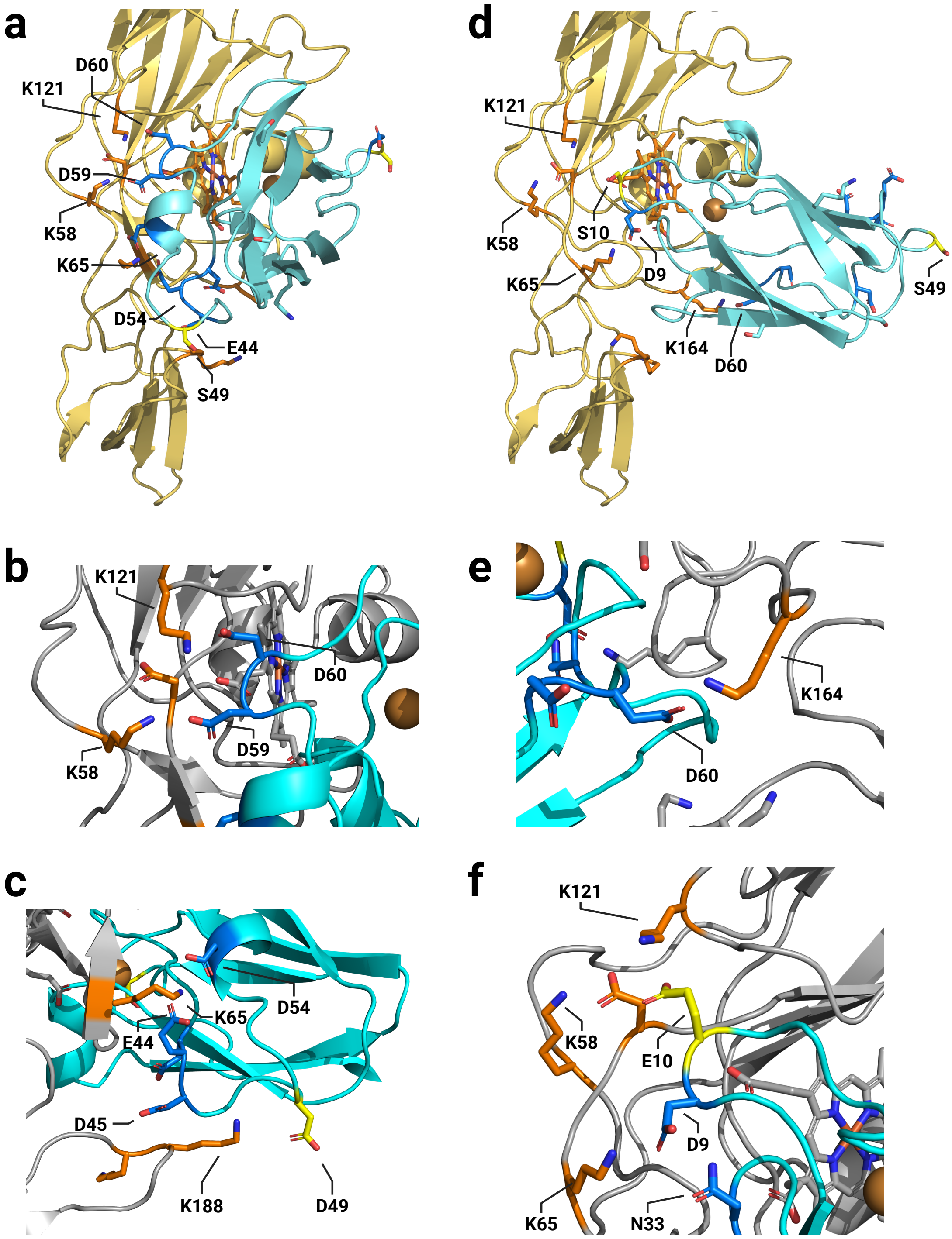

Supplementary Figure S3: Structural modelling of Cyt*f* and PC in complex. Illustration of the two models as presented in Figure 3. (**a**) This model takes into consideration the fact that algal PetA has an additional Lys at position 121, and that it was cross-linked to PC:D59/D60 (**b**). Additional interactions are observed between PC:D54 and PetA:K58/K65, PC:D43/E44 and PetA:K188/K65 (**c**). In addition, PC-S(D)49 seems to be in proximity of PetA:K188 and given a phosphorylation form might increase the stability of the complex formation. Core Cu-Fe distances are predicted to be 10.9 Å. (**d**) the second model takes into consideration a possible interaction between PC:D60 and PetA:K164 (**e**). This model also predicts an interaction between PetA:K121 and PC:S(E)10 and between PetA:K65 and PC:D9 (**f**). All models are available on Supplementary File S4. The illustration was generated using BioRender.com.
